# Towards better understanding of an industrial cell factory: investigating the feasibility of real-time metabolic flux analysis in *Pichia pastoris*

**DOI:** 10.1186/1475-2859-12-51

**Published:** 2013-05-21

**Authors:** Mariana L Fazenda, Joao ML Dias, Linda M Harvey, Alison Nordon, Ruan Edrada-Ebel, David LittleJohn, Brian McNeil

**Affiliations:** 1 Strathclyde Institute of Pharmacy and Biomedical Sciences (SIPBS), University of Strathclyde, 161 Cathedral St., Glasgow G4 0RE, UK; 2 REQUIMTE, Systems Biology & Engineering Group, DQ/FCT, Universidade Nova de Lisboa, Campus Caparica, Portugal; 3 WestCHEM, Department of Pure and Applied Chemistry and CPACT, University of Strathclyde, 295 Cathedral Street, Glasgow G1 1XL, UK

## Abstract

**Background:**

Novel analytical tools, which shorten the long and costly development cycles of biopharmaceuticals are essential. Metabolic flux analysis (MFA) shows great promise in improving our understanding of the metabolism of cell factories in bioreactors, but currently only provides information post-process using conventional *off-line* methods. MFA combined with real time multianalyte process monitoring techniques provides a valuable platform technology allowing real time insights into metabolic responses of cell factories in bioreactors. This could have a major impact in the bioprocessing industry, ultimately improving product consistency, productivity and shortening development cycles.

**Results:**

This is the first investigation using Near Infrared Spectroscopy (NIRS) *in situ* combined with metabolic flux modelling which is both a significant challenge and considerable extension of these techniques. We investigated the feasibility of our approach using the industrial workhorse *Pichia pastoris* in a simplified model system. A parental *P. pastoris* strain (*i.e.* which does not synthesize recombinant protein) was used to allow definition of distinct metabolic states focusing solely upon the prediction of intracellular fluxes in central carbon metabolism. Extracellular fluxes were determined using *off-line* conventional reference methods and *on-line* NIR predictions (calculated by multivariate analysis using the partial least squares algorithm, PLS). The results showed that the PLS-NIRS models for biomass and glycerol were accurate: correlation coefficients, *R*^*2*^, above 0.90 and the root mean square error of prediction, RMSEP, of 1.17 and 2.90 g/L, respectively. The analytical quality of the NIR models was demonstrated by direct comparison with the standard error of the laboratory (SEL), which showed that performance of the NIR models was suitable for quantifying biomass and glycerol for calculating extracellular metabolite rates and used as independent inputs for the MFA (RMSEP lower than 1.5 × SEL). Furthermore, the results for the MFA from both datasets passed consistency tests performed for each steady state, showing that the precision of *on-line* NIRS is equivalent to that obtained by the *off-line* measurements.

**Conclusions:**

The findings of this study show for the first time the potential of NIRS as an input generating for MFA models, contributing to the optimization of cell factory metabolism in real-time.

## Background

By 2007 the sales of all biopharmaceuticals totalled £60 billion representing 16% of the overall pharmaceutical industry and it is still growing, including the commercialisation of monoclonal antibodies (mABs) [1]. Biopharmaceuticals are a novel group of drugs which are revolutionising the treatment of serious causes of human ill health such as cancers, leukemias and degenerative illnesses. They are the most potent, the most complex and the most expensive drugs ever developed. The cost for biopharmaceuticals development can account for as much as 30% to 35% of the total cost of bringing a new drug to the market [2], so any reduction in the length of the development cycle of biopharmaceuticals could have a major impact on the overall drug economics. For this to happen, one of the most important factors is to improve our understanding of the metabolism of the cell factories (protein expression systems) during the key upstream step in the drug manufacturing process, the fermentation or cell culture step, in order to help ensure consistent drug quality, potency and half life. Although fermentation technology has made immense advances in recent years, our ability to understand and control in real time the metabolism of cell culture systems or microbial fermentations is still very limited [3].

Metabolic flux analysis (MFA) is considered a central pillar in modern systems biology for investigating metabolic networks [4]. The classic approach to MFA includes metabolic balancing (stoichiometric modelling) [5] or ^13^C-metabolic flux analysis using ^13^C-labeled substrates [6]. The application of MFA has been frequently shown to facilitate improved insights into cellular metabolism, and thus to enhance or increase production of desired products in both microbial [7-9] and cell culture systems [10] by identifying, for example, process bottlenecks and designing improved feeding strategies [4]. However, classic MFA approaches involve conventional *off-line* analytical methods (including GS-MS, LC-MS and NMR) or isotope tracing methods, which makes it complex, costly and time consuming. It is difficult to envisage such an approach lending itself to rapid *on-line* analysis [11]. By contrast, Goudar and co-authors [12] investigated the use of metabolite balancing to generate metabolic models of CHO cell cultures in quasi-real time. This approach showed much promise, but a large number of *off-line* measurements (27) were still necessary using various analytical methods, with results obtained post-process, highlighting the need for combining MFA with real time metabolite information [13]. If this were feasible, the integration of suitable real time analytical techniques could raise the utility of MFA to a new level offering enhanced real time metabolic understanding, which would really impact on clone selection capability, cell line development, rapid medium and process optimization in the development phase at lab scale, improved scale translation and finally to real time structured intervention in the metabolism of the cell factory (real time metabolic control). This would be a valuable tool in metabolomics and would represent a considerable addition to the toolkit available for industrial systems biology [14].

The obvious question which arises now is: Which real time, preferably *in situ*, techniques could be integrated into metabolite balancing to achieve this capability? Vibrational spectroscopy, driven by the Food and Drug Administration (FDA) initiative Process Analytical Technology (PAT) framework [15], has already shown considerable promise in the measurement of a range of analytes within both microbial and animal cell cultures both *at-line* (rapid off-line) or *on-line*, usually *in situ*[3,16]. Despite the evident attractions of vibrational spectroscopy, to date these techniques have not been used in metabolic modelling as they have been reported to have errors which are too high to be used in metabolic flux networks [12]. However, NIRS has a number of significant features which make it admirably suited to employment in such systems including, its non-destructive nature, rapidity of analysis (from a few seconds up to two minutes for a large scan number), the lower absorbances in the NIR region, which means it can be used without sample pre-treatment in matrices which are both highly absorbing and light scattering such as the typical fermentation fluid, the ease of sample presentation via steam sterilisable *in situ* probes and the potential to predict chemical and physical parameters from a single spectrum [16-18].

In the present study, we investigate the feasibility of establishing a robust platform technology for MFA by using a single probe, an *in situ* NIRS probe as a monitoring technique to predict near real-time intracellular metabolic fluxes in chemostat cultures of a *Pichia pastoris* strain. We chose *Pichia pastoris*, a methylotrophic yeast, as it is currently one of the most effective and versatile expression systems used in the biopharmaceutical industry for recombinant product production [19,20]. Chemostat cultures were used to allow a clearer definition of distinct metabolic states. We focused just on central carbon metabolism of a parental strain (*i.e.* that does not synthesize recombinant protein) to test the validity of our approach in a simplified model system. The findings of this study represent a significant step forward towards real-time metabolic control of cell factories and establishes a viable platform for using *on-line* vibrational spectroscopic measurements as inputs to MFA models in bioprocessing, replacing the use of *off-line* measurements (wet chemistry methods).

## Results and discussion

Central carbon metabolism in a chemostat culture for three glycerol-limited steady state *P. pastoris* cultures was analysed using the methods described in (Methods). The use of a parental strain of *P. pastoris* (not producing recombinant protein) simplified the system to allow the establishment of a framework for determination of intracellular metabolic fluxes by using NIR as an input-generating tool for MFA. This experimental system allowed the unambiguous examination of the link between changes in cell growth rate (determined by dilution rate) and the culture response, which would have been difficult in batch or fed-batch cultures. If the current study successfully proves that it is feasible to use such an approach combining real time NIRS measurements with metabolic flux modelling (MFA) in a simplified model organism, it will then be possible to take this research to the next level by examining the industrial relevance of this approach in fed-batch cultures of a protein secreting *Pichia* strain.

Dilution rates were varied between 0.05 to 0.15 h^-1^ in order to obtain three different states: low, medium and high dilution rates, designated as A, B and C (Table 1). The resulting metabolic states of the cells were subsequently assessed quantitatively via metabolic flux analysis with the extracellular metabolites rates calculated with both *off-line* measurements (reference) and *on-line* NIR predictions (section NIR modelling).

**Table 1:** **Glycerol-limited steady states (States A: low (0.05 h**^**-1**^**), B: medium (0.10 h**^**-1**^**) and C high (0.15 h**^**-1**^**) dilution rate) achieved in *Pichia pastoris *chemostat cultures**

	**A**	**B**	**C**
**Dilution rate, D (h**^**-1**^**)**	0.05	0.10	0.15
**CO**_**2**_**evolution, CER (mmol/L/h)**	21.34±0.42	24.82±0.36	30.24±0.44
**O**_**2**_**uptake, OUR (mmol/L/h)**	62.80±0.33	68.90±0.36	75.73±0.10
**Respiration quotient, RQ**	0.33±0.02	0.36±0.01	0.40±0.06
**Yield, Y**_**X/S**_**(g**_**DCW**_**/g**_**Gly**_**)**	0.63	0.67	0.67

### Fermentation data

**Figure 1:**
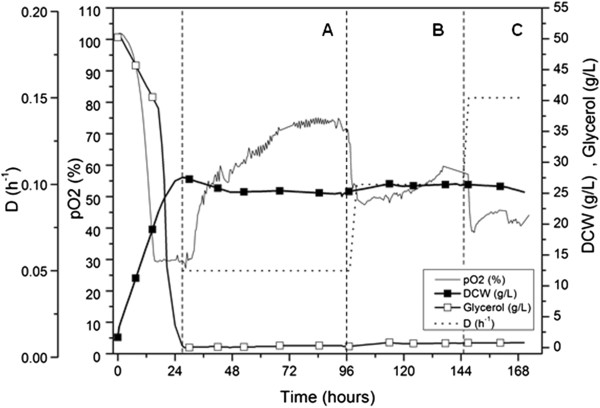
**Growth profile of *Pichia pastoris *chemostat culture: dilution rate (D), dissolved oxygen (pO2) percentage, dry cell weight (DCW) and glycerol concentrations over the course of the experiment.** States A, B and C: low (0.05 h^-1^), medium (0.10 h^-1^) and high (0.15 h^-1^) dilution rate, respectively.

The dry cell weight, glycerol, dissolved oxygen and dilution rate for the chemostat culture are shown in Figure 1. The cell physiology of each steady state was characterized by analysing the last four data points in each of the sections marked as A, B and C: low, medium and high specific growth rates, respectively. The preceding batch phase lasted approximately 24-27 h at which point the chemostat culture run was initiated by setting the dilution rate at 0.05 h^-1^. An increase of carbon evolution rate (CER) and oxygen uptake rate (OUR) with dilution rate (from state A to C) were observed (Table 1), however biomass yields increased only from state A to B, with negligible differences between medium and high dilution rate (state B and C).

### NIR modelling

#### Model development

**Figure 2:**
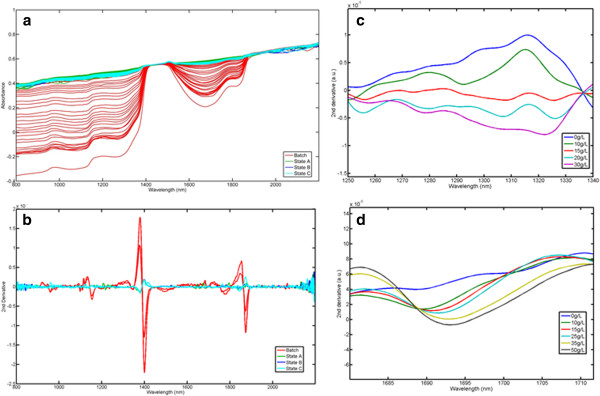
**(a) Raw and (b) Pre-processed (2nd derivative) NIR spectra of *Pichia pastoris *chemostat cultures c) 2nd derivative spectra of 0 and 30 gL-1 of aqueous solutions of Biomass from 1250-1340 nm and (d) 2nd derivative spectra of 0 and 50 gL-1 of aqueous solutions of glycerol from 1680-1715 nm.** States A, B and C: low (0.05 h^-1^), medium (0.10 h^-1^) and high (0.15 h^-1^) dilution rate, respectively.

The raw spectra of the chemostat culture of *P. pastoris* (Figure 2a) show changes in the spectral baseline as well as in the signal intensity, becoming closer to detector saturation with process time. Any other spectral changes directly linked to the analytes of interest in these raw spectra that are relevant for the calibration model are difficult to visualise and so it is crucial to use appropriate pre-processing techniques. Savitzky-Golay second derivative and mean centering were applied in the model development cycle to help remove unimportant baseline signals from the samples and extract relevant “hidden” information. In the second derivative data, absorbance maxima are converted to minima that are enveloped by positive side lobes [17]. In addition, spectral data bandwidth is sharply reduced allowing for resolution of overlapping peaks, and the baseline differences between spectra are largely eliminated. After removing the dominant water peaks (1400 and 1900 nm) and the spectral region above 2000 nm (not usable when using silica fibre optic probes due to the noise of the fibres having an adverse effect on the spectra [18]), spectral changes with process time were identified in the second (1050 – 1650 nm) and first overtone (1450 – 2050 nm) regions. For biomass, -CH absorption bands were identified in the 1250 –1350 nm region, while for glycerol a broader region was chosen (1500-1800 nm), which includes the –OH stretch band and other -CH second overtone bands [21]. As can be seen in Figure 2b it is possible to see the progress of the fermentation process from the derivatized spectra, for example in the region of 1500-1800 nm, where glycerol is absorbing, the most negative peak corresponds to the batch phase of the process, with process time and as the glycerol concentration decreases the peak becomes less negative due to glycerol consumption by the culture. In order to assess the selected wavelength regions with respect to the different analytes monitored, aqueous solutions of both biomass and glycerol were scanned [22]. Figure 2c and d show the relevant regions for biomass and glycerol in aqueous solutions, respectively. The wavelength regions used in the actual PLS models were broader to include process variations in the spectral information over a wider wavelength window resulting in better results.

Once the spectra were pre-processed, it was necessary to identify the time periods of interest for each analyte. Due to the very low (limiting) concentration of glycerol (<1 g/L) and only slight changes in the concentration of biomass during the continuous phase, models developed based solely in this phase failed validation and therefore, the correspondent predictions could not be used for the metabolic flux analysis (data not shown). Because PLS-NIRS models are based on the amount of variance captured during the calibration procedure: the more changes in the process it includes, the more robust the models will be. These changes can be: inherent variations of analyte levels, accumulation profiles, spectral matrix differences and interfering absorptions [17]. If the concentration range being modelled is very limited, the PLS algorithm is not able to provide good predictions, and PLS models fail validation, as seen when only the chemostat phase was included. Therefore spectra from both the batch and chemostat dynamic phase (the period between steady states) were included to capture as much as possible the inherent variations in analyte levels, accumulation profiles, spectral matrix differences, and interfering absorptions. During the first phase of the process (batch) the glycerol concentration rapidly changed (from 40 g/L to 0 g/L) as it is rapidly being consumed for formation of biomass. This will ensure that the quantification of analytes from the NIRS models are representative and include enough variation in sample properties so these models could be potentially used in future fermentation runs [17]. The enhanced range of analyte concentrations and spectral variation led to more robust PLS models capable of predicting accurately the analyte concentrations in this study (Table 2).

**Table 2:** **Near Infrared calibration models and validation for glycerol and biomass in *Pichia pastoris *chemostat cultures**

**Model**	**Range (g/L)**	**λ (nm)**	**LV**	**Calibration**		**Cross-validation**		**External validation**		**RMSEP RMSECV**
				**RMSEC (g/L)**	**R**^**2**^	**RMSECV (g/L)**	**R**^**2**^	**RMSEP (g/L)**	**R**^**2**^	
**Glycerol**	0-40	1500-1800	3	1.70	0.98	3.38	0.95	2.90	0.95	0.86
**Biomass**	0-30	1250-1350	5	0.48	0.97	0.93	0.91	1.17	0.94	1.26

λ: wavelength; *LV*: number of latent variables; *RMSE*: root-mean square error; *R*^*2*^: correlation coefficient.

#### Model validation

**Figure 3:**
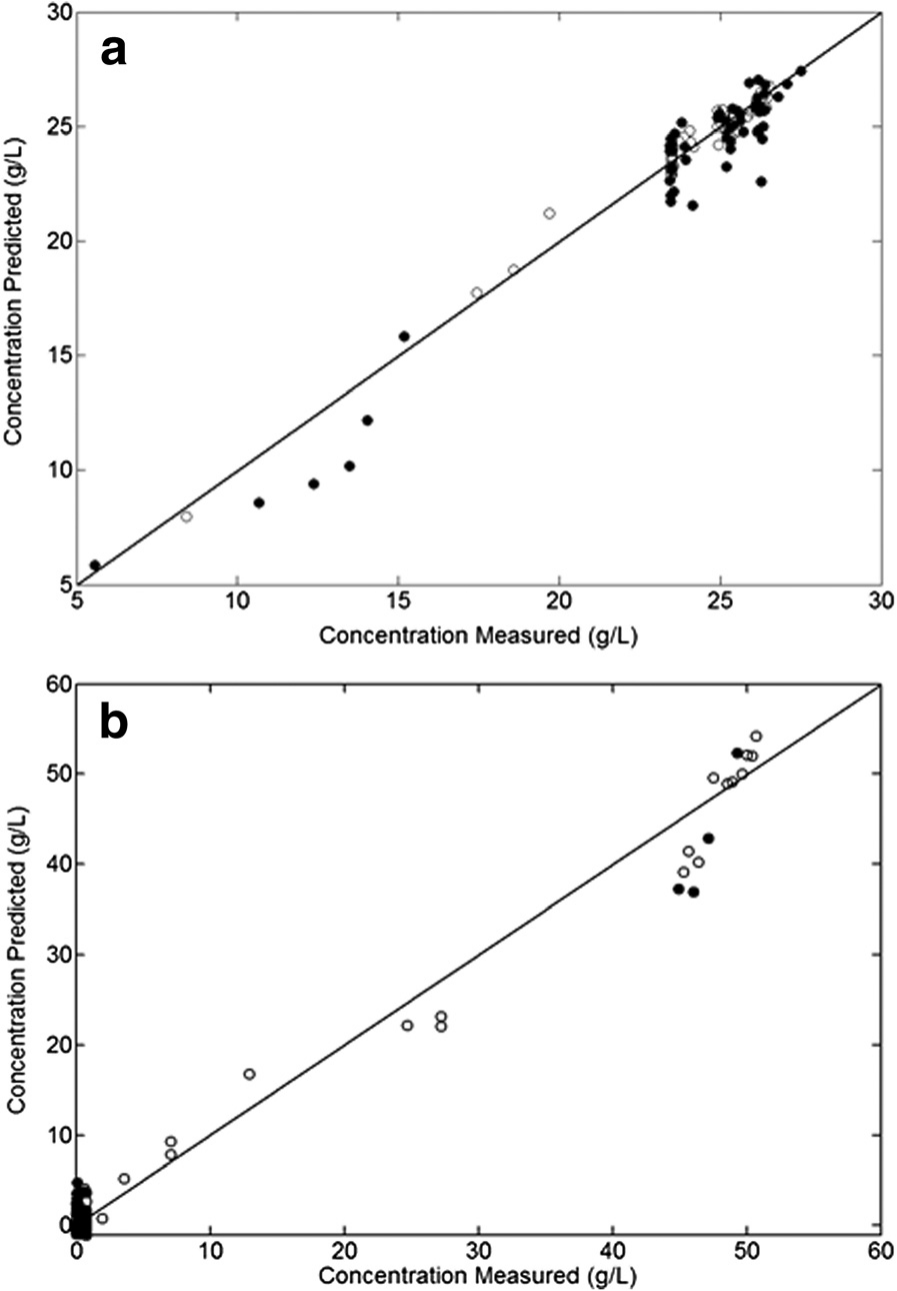
**(a) Raw and (b) Pre-processed (2nd derivative) NIR spectra of Pichia pastoris chemostat cultures. States A, B and C: low (0.05 h-1), medium (0.10 h-1) and high (0.15 h-1) dilution rate, respectively.** Blank circles represent samples from the calibration (training) set and filled circles represent samples from the validation (test) set.

From the calibration statistics (Table 2), both glycerol and biomass PLS models performed reasonably well, with *R*^*2*^*-values* above 0.90 or above for both calibration and external validation, indicating a good fit between the measured and predicted data. The RMSEP/RMSECV ratios were very close to one, indicating no significant differences between the performance of the calibration and validation models and so robustness in the face of process and analyte variation. This result is a good indicator of how in practical terms the models would behave when exposed to process-to-process variability seen in fermentation processes. Figure 3 shows the concentration correlation plots after internal and external validation of the biomass and glycerol calibration model. The models predictive ability in the fermentation broth is high as all the samples are distributed along the y = x line.

According to the SEL values shown in Table 3, the RMSEP values for both biomass and glycerol models are lower than 1.5 × SEL, which indicates that the performance of the NIR models is suitable for quantifying biomass and glycerol as the basis for calculating extracellular metabolite rates to be used in the MFA model [23,24]. NIR has never been used for such real-time metabolic modelling, mainly because it has been in the past associated with high errors relative to the concentrations of key analytes [25]. Goudar *et al.* (2006) [12] considered the errors reported in two papers on NIR and MIR to be unacceptably high (above 20%) [25,26], and for that reason they suggested inappropriate the use of such spectroscopic techniques as a replacement to the current largely *off-line* methods used. The present authors have intensively studied the application of NIRS to fermentation and cell culture systems to predict a number of different analytes simultaneously (from biomass to different substrates and types of products, including proteins) [18,27,28] and the reported errors in these studies were significantly lower than those reported above. In the present investigation we show that the validation errors (RMSEP) for biomass and glycerol are less than 10% of the concentration range of the analytes, and in terms of sensitivity, NIRS was comparable to the reference methods used (Table 3). Furthermore, both biomass and glycerol NIR models yielded *R*^*2*^, RMSECV and RMSEP values close to other published studies using *on-line* NIR probes [29,30]. This is within the concentration range Goudar et al. (2006) suggest as acceptable for analyte quantification [12]. Thus, the major objection in principle to the use of NIRS to predict extracellular fluxes accurately enough to use these values as inputs to MFA models is shown not to be valid. As demonstrated here, careful calibration procedures, taking into account appropriate choices wavelength selection protocols, can be effectively used to generate effective NIR analyte models with acceptable errors simultaneously for more than one analyte in near real-time. It is also important to note that NIR models were built based on spectral samples taken every 30 min (the sample interval can be decreased if necessary), with no-sample preparation and no additional cost, as opposed to the time-consuming and costly reference methods used.

**Table 3:** Method qualification results for reference method and for NIR

**Analyte**	**Method**	**Range**	**Reference method**	
			**SEL**	**SD**_**mean**_
**Glycerol**	Enzymatic assay	0 – 40	2.37	2.20
**Biomass**	Gravimetry	0 – 30	0.79	0.56

*SEL*: standard error of laboratory; *SD*_*mean*_: mean standard deviation of replicate measurements.

All results are shown in g/L. The method and the concentration range of each analyte used in the PLS models are also shown.

There are other sensor systems used in bioprocessing, but their applicability is still very limited with respect to *on-line* and in-situ measurements either due to specific aseptic conditions requirements, low or single number of measured analytes, drift and other rather low physiological relevance [31]. Raman and Mid-Infrared spectroscopy are strong candidates for multivariate analysis in fermentation systems, but their in-situ technology is still under development [32,33]. Fluorescence based methods, dielectric spectroscopy, flow cytometry are optical methods commonly used to determine biomass, one of the most challenging as well as important measurements in the bioreactor cultivation of live cells. But again they are influenced either by the culture conditions (*eg.* stirring, aeration, broth conductivity, pH and viscosity), or the in-situ probes are still in development, or are dependent upon the cell type and size, and not always are able to measure multiple fermentation parameters [31,34].

NIR probes offer a high-quality signal from a bioprocess, they are physically robust instruments compared to the aforementioned sensors, suited for industrial manufacturing processes, being able to monitor multiple analytes simultaneously, avoiding the need to use multiple *on-line* sensors, which is often a limiting step in a fermenter set-up. NIRS technology is applicable to most fermentation expression systems, to batch and fed-batch systems, being able to monitor not only biomass and glycerol (as in this study) but also other metabolites such as glutamate, glutamine, ammonium, alcohols, proteins, organic acids, etc. [35] with the aid of multivariate data analysis as well as capturing physical process changes (viscosity, temperature changes, contaminations, system failures), an advantage for process control, in particularly in the production of biopharmaceutical [16,35].

Thus, the NIRS constitutes one of the most favourable *on-line in situ* technology to form the basis for near real-time measurements of multiple inputs to metabolic flux modelling of cell factories in bioprocessing. The findings presented here constitute the first step part of a structured multidisciplinary research programme, to be applied for modelling cell factories (such as protein expression systems) using microbial and cell line approaches as well as dynamic (fed-batch) fermentation processes. Transferring this technology from the simplified system used here to an industrial process will involve addressing significant new challenges. These include: first, high cell density and associated very short process time constants; second, increased metabolic complexity. Regarding the first point, it has been demonstrated that NIR can deal with measurements in high cell density systems [1-3], including *Pichia*, especially where the medium is soluble (as here). *In situ* spectroscopy is also very fast (seconds per spectral acquisition). As to increased metabolic complexity, there are existing published metabolic models of *Pichia* that can be built upon [4,5]. Due to published mis-conceptions on error in IRs measurements (discussed above), it was first of all necessary to establish the feasibility of applying NIR as an input measurement system for MFA in a simplified system. In this study we have done so.

### Metabolic flux analysis

#### Network properties

**Figure 4:**
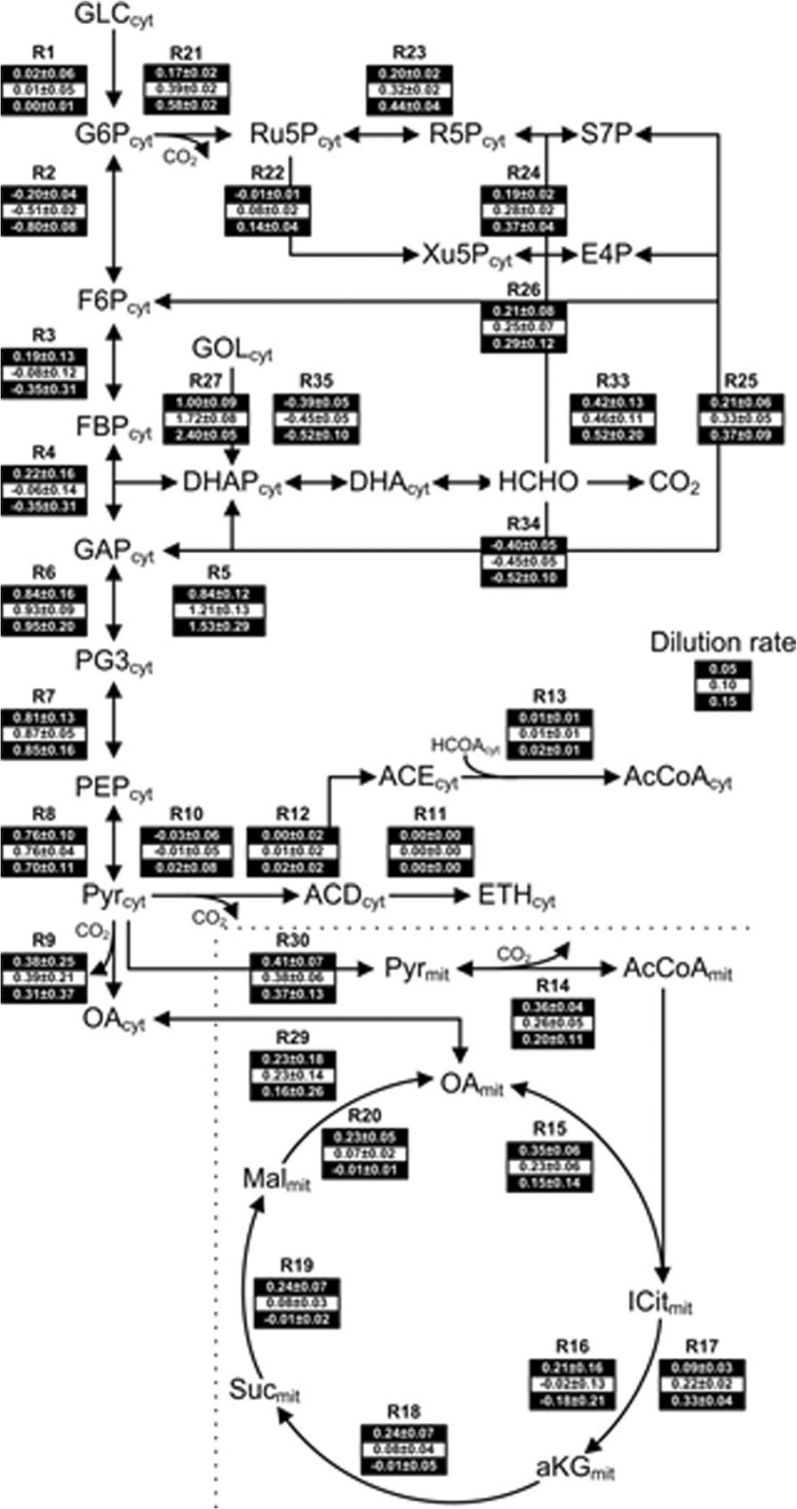
**Metabolic flux distribution in *Pichia pastoris *and respective intracellular flux NIR predictions in glycerol-limited chemostat at low (state A=0.05 h**^**-1**^**), medium (state B=0.10 h**^**-1**^**) and high (state C=0.15 h**^**-1**^**) dilution rates.** The fluxes for each reaction in the network corresponding to a D of 0.05, 0.10 and 0.15 h^-1^ are given from top to bottom, respectively (mmol/gDCW/h). Metabolites names are displayed in bold: GLC = glucose; G6P = glucose-6-phosphate; F6P = fructose-6-phosphate; FBP = fructose-1,6-biphosphate; GAP = glyceraldehyde-3-phosphate; DHAP = dihydroxyacetone phosphate; DHA = dihydroxyacetone; HCHO = formaldehyde; CO2 = carbon dioxide; GOL = glycerol; RU5P = ribulose-5-phosphate, XU5P = xylulose-5-phosphate; R5P = ribose-5-phosphate; S7P = sedoheptulose-7-phosphate; E4P = erytrose-4-phosphate; PG3 = 3-phosphoglycerate; PEP = phosphoenolpyruvate; PYR = pyruvate; ACD = acetaldehyde; ETH = ethanol; AcCoA = acetyl CoA; ACE = acetate; OA = oxaloacetate; ICIT = citrate; aKG = alpha-ketoglutarate; Suc = succinate; Mal = malate; (cyt) = cytosol; (mit) = mitochondria.

A simplified biochemical reaction network was adopted here for *P. pastoris* (adapted from [36]), and is illustrated in Figure 4. Only the central carbon metabolism was considered, including reactions that are important for producing biomass and energy (glycolysis, tricarboxylic acid cycle - TCA, pentose phosphate – PPP, and fermentative pathways). It includes a total of 44 reactions and 45 compounds, and the balanced growth condition can be applied to 36 internal metabolites, resulting in a 36 × 44 stoichiometric matrix with 8 degrees of freedom (*m-n*); the matrix and the list of reactions is given in the Additional file 1. The overall pathway was simplified by grouping some reactions into single ones without any loss of accuracy of representation [36]. The aim is to use a model with which is possible to investigate applying NIRS as a novel tools able to improve process monitoring and control in real time. The intracellular fluxes were calculated from the extracellular rates using the two independent datasets: the *off-line* and *on-line* (NIRS) measurements (section Extracellular flux rates); and the stoichiometric model described above. The stoichiometric model was constrained by the extracellular metabolites, i.e. uptake/production rates for O_2_, CO_2_, glycerol and biomass and the production rates for ethanol, citrate and pyruvate, which based on HPLC analysis were not detected and therefore considered null (results not shown). The following major assumptions were used in the model: (i) the biomass synthesis reaction (r44) was considered constant under the different process conditions and it was based on the macromolecular composition of *P. pastoris* from [37]; (ii) the glyoxylate cycle was considered inactive [38].

#### Extracellular flux rates

**Figure 5:**
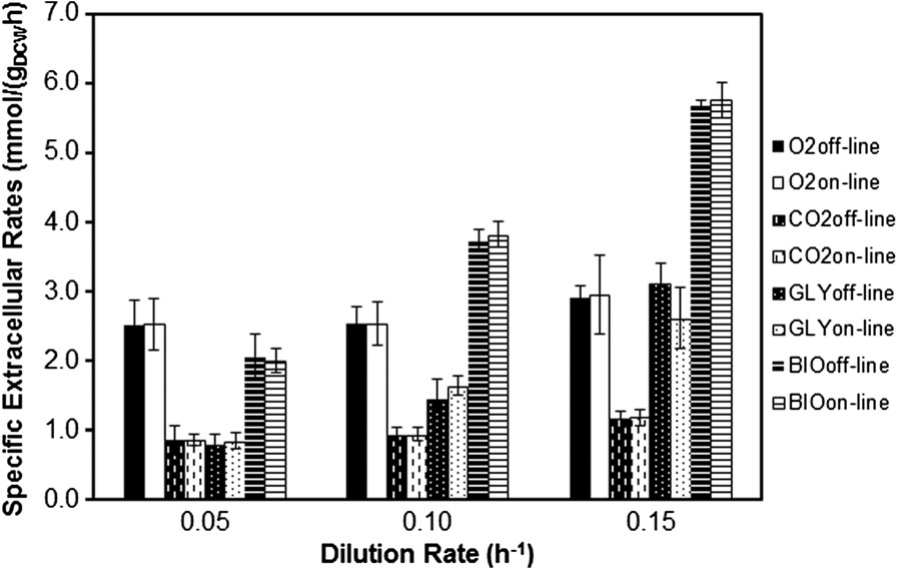
**Calculated Extracellular Rates (mmol/gDCW/h) for Oxygen, Carbon Dioxide, Glycerol and Biomass at low (state A=0.05 h**^**-1**^**), medium (state B=0.10 h**^**-1**^**) and high (state C=0.15 h**^**-1**^**) Dilution Rates in *Pichia pastoris *chemostat culture using both off-line and on-line (NIR) measurements.**

The concentration of both glycerol and biomass calculated from both the *off-line* measurements and *on-line* NIR predictions were used for the calculation of the specific extracellular rates shown in Figure 5. The errors presented are the propagated errors [39] which include both metabolite measurement errors and also biomass errors. As D increases, an increase of 3, 1.5 and 3-fold, respectively, of the glycerol consumption and both CO_2_ and biomass specific production rates were observed (from both *off-line* and *on-line* calculations). However, the changes relative to the oxygen consumption rate were not significantly different between each state. Using a *t-test* statistical analysis between *off-line* and *on-line* results for each dilution rate and each of the extracellular rates determined, it was clear that there were no significant differences between the *on-line* and *off-line* extracellular rates (p<0.001). The extracellular rates were then used to compute intracellular metabolic fluxes for state A to C using Equation 5. The predictions from NIR extracellular rates were very close to the rates derived from the *off-line* assays, as expected from the input rates. For that reason, only the intracellular fluxes predicted by the *on-line* NIR measurements for the *P. pastoris* chemostat cultures at different dilution rates are shown in Figure 4 (the intracellular fluxes predicted by *off-line* measurements can be found in Additional file 1). Negative values signify that the reaction is operating in reverse direction. Key metabolic fluxes and pathways are discussed below.

#### Consistency test

The values obtained for the consistency index *h* for each steady state (NIR and experimental data) were lower than the corresponding χ^2^ (chi-square) values for a 95% confidence interval (3.84) and the one redundant equation (*i.e.* consistency test degrees of freedom), as seen in Table 4. This shows that the *off-line* and *on-line* data are both consistent with the pseudo-steady assumption and also the assumed biochemistry, and that there are no systematic or gross measurement errors. The results from statistical analysis on the extracellular rates and the consistency test show that NIR spectrometry can be used as an *on-line* tool in fermentation systems to determine real-time intracellular metabolite fluxes.

**Table 4:** Consistency test validation results

**State**	**DF**	***h***_***exp***_	***h***_***NIR***_	**χ**^**2**^	**Pass/fail**
**A**	1	2.50	2.29	3.84	Pass
**B**	1	3.34	1.54	3.84	Pass
**C**	1	2.68	0.00	3.84	Pass

#### Effect of dilution rate on central carbon metabolism of *P. pastoris* cells

Figure 4 shows the predicted *on-line* NIR intracellular carbon fluxes observed in the chemostat culture of *P. pastoris* under different dilution rates. In some instances, the term ‘relative flux’ will be used to describe fluxes normalized to the specific glycerol uptake rate (i.e. r_*x*_/r_40,_ where r_x_ is the reaction number of the flux reaction used, and r_40_ the flux reaction for glycerol consumption) and to allow a direct comparison among the different states investigated (state A to C), this type of terminology is widely used in the context of MFA [40,41].

##### Flux distribution in glycolysis/gluconeogenesis and PPP

Glyceraldehyde-3-phosphate (GAP) links the glycolysis/gluconeogenesis pathways with the methanol and glycerol uptake pathways in the *P. pastoris* metabolic network. At all dilution rates the majority of glycerol is funnelled into the glycolytic pathway (r_6_), and much less towards gluconeogenesis (r_4_) (Figure 4). However, with the increased dilution rate the relative fluxes of glycerol to glycolysis decrease (r_6_/r_40_: from 86% to 40%, from state A to C, respectively), while an increase is seen from gluconeogenesis towards the PPP (r_4_/r_40_: from 22% to −15%, from state A to C, respectively – note that this reaction is reversible). The increase of the relative flux through the PPP (r_21_/r_40_) coincided with an increase in biomass yield (from 0.63 g_DCW_g^-1^_Glycerol_ at low dilution rate to 0.67 g_DCW_g^-1^_Glycerol_ at 0.15 h^-1^). Furthermore, the carbon flux through the PPP (r_21_) that re-entered glycolysis at the level of fructose-6-phosphate (F6P) and glyceraldeheyde-3-phosphate (GAP) (r_26_) was at least 50% less at higher dilution rates (states B and C) than at lower dilution rate (state A). This indicates that the PPP is being used for NADPH generation to support the production of biomass. It is known that the oxidative part of the PPP (r_21_) is considered to be the major NADPH regenerating reaction in yeast and is driven by the demand for biomass synthesis, and in fact here it showed the highest flux of all the PPP reactions (Figure 4). Heyland (2010) [42] suggested a similar findings to ours: “as NADPH is used as an electron donor during biomass synthesis and the glucose-6-P and 6-P-gluconate dehydrogenases of the PPP pathway are the major NADPH regenerating reactions in yeast (r21), the flux increase through the PPP pathway might be a direct effect of the higher biomass yield, as suggested previously by Blank *et al.* (2005) [43]”. The higher biomass yield observed here at higher dilution rate (state C) is likely to be a result of the flux increase through the PPP.

##### Flux distribution in TCA

In all steady states (from A to C) the cells exhibited a respiratory metabolism, as the fluxes from respiro-fermentative metabolism (from r_10_ to r_13_) were zero (Figure 4), typical of aerobic culture conditions. This supports the view that *P. pastoris* cells operate to produce biomass with very little or no by-product formation when they are not oxygen limited and are growing on glycerol [40,44]. On the other hand, it was observed that the flux entering the TCA cycle from the pyruvate node (r_30_) was approximately the same at all dilution rates. This had consequences in terms of the TCA cycle: from alpha-ketoglutarate (r_18_) onwards the TCA cycle activity significantly decreased at the highest dilution rates (state B and C).

##### Flux distribution and biomass synthesis

Alpha-ketoglutarate (aKG) represents a key metabolite linking the entry and exit of carbon sources from the TCA cycle to pathways involved in amino acid metabolism for biomass synthesis [8,42]. At higher dilution rates the energy requirements are fulfilled without the complete TCA activity and most of the carbon flux in the TCA is channelled towards synthesis of the biomass precursors from aKG. This is supported by the decrease in the fluxes in the reactions r_18_-r_20_, as the remaining TCA metabolites (succinate and malate) are not involved in the synthesis of biomass. It is possible that the TCA cycle is operating at its maximum capacity when the specific growth rate is high as it has been proposed in other studies [41,45], and therefore the fluxes of the TCA metabolites are limited at higher dilution rates, as the emphasis moves towards rapid protein synthesis to generate new cell mass driven by the high feed rates (states B and C). Under such conditions of stress, cells look to the PPP and gluconeogenesis pathways as an alternative source of energy and building blocks for cell growth. The increase seen here in PPP fluxes would logically be associated with the increase of other cell building blocks, such as DNA and RNA that are mainly produced through these pathways. Similarly, in chemostat cultures of *S. cerevisiae* it has been reported that a decrease in the cell protein content at higher dilution rate may be related to a significant decrease in the TCA cycle fluxes [46]. This suggests that *P. pastoris* cells grown at high dilution rates might need nutrient supplementation to maintain high rates of protein synthesis, while at lower dilution rates the cultures would be more self-sufficient. This effect would be more pronounced if a heterologous protein were being expressed at high levels, as seen in [37,41]. In the present study as specific growth rate is pushed higher increased need for biosynthetic intermediates leads to the situation noted here, where the TCA intermediates are increasingly diverted to intracellular metabolites (aKG) linked to biomass synthesis.

## Conclusions

The applicability of NIRS with advanced process modelling to define real-time metabolic fluxes in the industrially relevant cell factory *P. pastoris* is reported herein. A single in-situ NIR probe was used to predict biomass and glycerol consumption rates in near real time. Focusing only on central carbon metabolism of these cultures enabled us to assess the viability of this novel approach in light of previously published objections which indicated that this approach was not appropriate.

The results of the MFA showed that the NIR predictions were equivalent to the off-line reference methods, thus showing the potential of NIRS to be used in real-time MFA. The implementation of NIRS-MFA has potential utility in mainstream biotechnology industries, in particular, it expands our ability to monitor and control the metabolism of a key cell factory in real time using a low cost, robust multivariate monitoring technology as opposed to the current costly, complex and time consuming methods. Advances in metabolic flux modelling are essential to complement other rapidly expanding applications in biotechnological techniques, such as transcriptomics and proteomics, in the development of novel biopharmaceuticals. Further work using more complex systems e.g. protein producing strains where the metabolic network will be expanded to include protein synthesis pathways, is on-going. This substantially extends the challenges encountered in this study and permits a systematic evaluation of this potential platform technology’s industrial relevance and utility.

## Methods

### Strain and media

A *Pichia pastoris* strain CBS7434 MutS was supplied by Ingenza Ltd (UK). The yeast strain was kept as frozen stock cultures in Yeast Nitrogen Base (YNB) medium (34 gL^-1^ YNB) at −80°C. Inocula were prepared from 1 ml of the frozen culture sample which had grown on a 2-L shake flask in 600 ml of standard BMGY medium, containing 1% Yeast Extract, 2% Mycological Peptone, 1% Glycerol, 1.34% YNB, 100 mM Potassium phosphate (pH 6.0) and 4×10^-5^% Biotin; This was grown at 30°C, 250 rpm, for 20–24 hours until an OD_600_ of approximately 2.5 was reached. Finally the contents of the flask were transferred to the bioreactor to create an inoculum concentration of 10% (v/v). The chemostat culture contained per liter: 40 g of glycerol; 26.7 mL H_3_PO_4_ (85%); 14.9 g MgSO_4_⋅7H_2_O; 0.93 g CaSO_4_; 18.2 g K_2_SO_4_; 4.13 g KOH; 4.35 mL of PTM_1_ salt solution; and 0.1 mL antifoam.

### Chemostat cultivation

The reactor used was a 15-l (total volume) stainless steel bioreactor (BIOSTAT C.-DCU, B. Braun Biotech International, Switzerland). The cultivation was carried out at a temperature of 30.0±0.1°C and a pH of 5.5±0.1 (maintained by automatic addition of 24% NH_4_OH). The dissolved oxygen concentration (DO) was maintained above 30% of saturation by a cascade controlling system (maintained by an agitation rate of 300–1200 rpm and aeration rate 1–3 vvm). Exit gases (O_2_ and CO_2_) were measured as % using a digital gas analyzer TANDEM Pro (Applikon Biotechnology Ltd, Gloucestershire, UK). Real time values of pH, DO, agitation speed, temperature, air-flow rate, O_2_ and CO_2_ were recorded automatically by the bioreactor software MFCS DA (Sartorius, UK). After 24 h, the feed and waste pumps were started to initiate chemostat cultivation. Following three to four residence times, time invariance of the following variables was assumed to indicate a steady state: optical density, glycerol concentration, oxygen uptake and carbon evolution rate, OUR and CER respectively. Three steady states were achieved with the following dilution rates: A=0.05 h^-1^, B=0.10 h^-1^ and C=0.15 h^-1^. Knowing that the maximum specific growth rate, μ_max_, of *P. pastoris* on excess glycerol is 0.17 h^-1^[44], states A, B and C were considered: low, medium and high dilution rate, respectively.

### Sampling and *Off-line* measurements

Samples were taken approximately every 4 hours over the cultivation process and analysed as described below. Biomass was estimated by gravimetric difference as dry cell weight (DCW): 5-ml of culture fluid was filtered onto a pre-dried, pre-weighed 0.2 μm filter (Whatman, Maidstone, UK). Cells were washed with 2×5 ml of sterile water and the filter dried to a constant weight in an oven (105°C for 24 h). Glycerol determination was carried out using a Boehringer Mannheim Glycerol enzymatic kit (148–270, Lewes, UK) at 340 nm.

### NIR

Spectra were acquired with a dual beam NIR process spectrometer (Foss- NIRSystems Inc., Silver Spring, MD, model XDS) using a transflectance probe submerged in the bioreactor with a gap of 0.5 mm resulting in an effective path length of 1 mm was sufficient to acquire reasonable spectra. Spectral measurements were referenced against a NIST traceable reference material (serial number R99P0079). Due to the nature of the reference and the design of the probe the referencing procedure was carried out with a reflectance probe (Foss NIRsystems, Maryland, USA). A correction factor was then applied to compensate for the differences in the acquired spectra from the reflectance probe, used in the instrument calibration procedure, and the transflectance probe that was utilised for spectroscopic measurements. The probe was mounted in one of the side sampling ports of the bioreactor. As these ports also housed the pH and dissolved oxygen probes, any sample measurements made should have been representative of the whole reactor contents and would not have had a significant impact on the mixing efficiency of the reactor. Spectral measurements (X data) were an average of 32 scans, taken every 30 minutes over the NIR range of 800–2200 nm.

#### Data analysis and calibration development

Spectral collection was performed using VISION (version 3.0, Foss NIRSystems) and calculations were carried out using Matlab version 7.12 (2011a) (MathWorks, Natick, MA) and the PLS Toolbox version 6.5.1 (Eigenvector Research, Manson, WA). Multivariate calibration models were developed with the PLS algorithm. All the spectra were mean-centered, and second-order Savitzky-Golay (SG) derivatives were applied (filter width of 33 data points and a second-order polynomial fit) before development of the calibration models. The performance of developed models was assessed by global analysis of the root mean square errors of cross-validation (RMSECV) and prediction (RMSEP), latent variable (*lv*) number, and the respective correlation coefficient, R^2^, between the predicted and measured values for both calibration and validation sets [47]. Biomass and Glycerol concentration data (Y data) determined from the *off-line* measurements were interpolated using the *interp1* command (linear interpolation) in Matlab and matched to the NIR spectral data. A random number table was used to divide the data into calibration (85 samples) and validation sets (30 samples), so that the model could be externally validated in the absence of more samples. To aid in gaining an analytical basis for the model development, NIR spectra of aqueous solutions of glycerol (0–50 g/l) and aqueous suspensions of biomass (0–30 g/l), all adjusted to the bioprocess pH, were also prepared and scanned (Figure 2c and d).

#### Reference method qualification

Reference method qualification was accessed using the statistic standard error of laboratory (SEL). SEL is defined as the standard error of variance between replicates analyzed by the reference method and was calculated using Equation 1 [23]. 16 samples were analysed in duplicate for each analytical method (see section Sampling and *Off-line* measurements). Accuracy can be determined by agreement between the RMSEP and SEL. As a rule [23,24], considering that the *R*^*2*^ and the RMSEP indicate the precision achieved in the NIR calibration, *R*^*2*^ values higher than 0.90 indicate excellent precision, as well as RMSEP values lower than 1.5 × SEL. *R*^*2*^ values between 0.70 and 0.90 mean good precision, as do the RMSECV values between 2–3 × SEL. While, models with *R*^*2*^ values lower than 0.70 are only suitable for qualitative purposes, allowing distinction between low, medium and high values for the measured parameter being analysed.

(1) $$\mathrm{SEL}=\sqrt{\frac{\sum_{j=1}^{N}\sum_{i=1}^{r}{\left(y_{\mathrm{iy}}-\bar{y_{i}}\right)}^{2}}{N\left(r-1\right)}}$$

where *y*_*ij*_ is the i^th^ replicate of the j^th^ sample, ${\bar{y}}_{1}$ is the mean value of all replicates of the j^th^ sample, *r* is the number of replicates and *N* is the total number of samples.

### Estimation of specific extracellular rates

The specific growth rate was determined from the mass balances in the fermenter, resulting in

(2) $$\mu =\frac{F}{V}+\frac{1}{X}\frac{\mathrm{dX}}{\mathrm{dt}}$$

where μ is the specific growth rate (h^-1^), *F* the flow rate (L/h), *V* the fermenter volume (L), *X* the biomass concentration (mol/L), and *t* is time (hours). Accordingly, specific uptake or production rates for glycerol, O_2_ (OUR) and CO_2_ (CER) were calculated based on:

(3) $$q=\frac{F\left(C_{\mathrm{in}}-C_{\mathrm{out}}\right)}{\mathrm{VX}}$$

where *q* is the specific uptake or production rate (mmol/(g_DCW_h)), while *C*_*in*_ and *C*_*out*_ are the reactor inlet and outlet concentrations (mol/L) of the nutrients or metabolites. The extracellular rates were determined from Equations 2 and 3 using six separate data points from the end of each dilution state (A, B and C) and averaged to obtain a single value for each state. The biomass concentration was determined either by the *off-line* method described in 4.5 or by the *on-line* NIR predictions.

### Metabolic flux analysis

Assuming a pseudo-steady state approximation (PSS), the material balance equation for the intracellular metabolites [48] can be written as

(4) $$\frac{\mathrm{dc}\left(t\right)}{\mathrm{dt}}=0=A*r\Rightarrow 0=A*r=A_{n}r_{n}+A_{b}r_{b}$$

The intracellular fluxes were calculated based on the material balance model expressed in matrix notation where *A* is the matrix of stoichiometric coefficients consisting of *m* rows corresponding to the intracellular metabolites and *n* columns corresponding to the number of metabolic reactions (Additional file 1). The vector *r* contains the net reaction fluxes (mmol/(g_DCW_h)) and vector *c* denotes the concentrations of the intracellular rates. *A* and *r* were split into the unknown (*A*_*n*_*and r*_*n*_) and known (*A*_*b*_*and r*_*b*_) matrices and vectors of stoichiometric coefficients and rates, respectively. The known and unknown rates correspond to the extracellular and intracellular metabolic fluxes, respectively. The intracellular metabolite fluxes can be determined from Equation 5 using simple matrix inversion if *A* is square (*m* = *n*). If *A* is not a square matrix, the system becomes redundant and it is valuable to include the surplus information to check the consistency of the data and the assumed biochemistry. In this case *m* >*n* and the fluxes can be determined via the method of weighted least squares [49]:

(5) $$r_{n}\;=\;-{\left(A_{n}^{T}\Psi A_{n}\right)}^{-1}A_{n}^{T}\Psi^{-1}r_{b}$$

where ψ is the variance-covariance matrix of the extracellular fluxes calculated directly from the measurements. Once the intracellular rates were estimated, the consistency of the metabolic fluxes to the measurements was estimated by calculating the consistency index, *h* given by:

(6) $$h=e'\;*p_{\mathrm{inv}}\left(J\right)*e$$

With *J,* the variance-covariance matrix of the vector of residuals (Equation 9), *p*_*inv*_(*J*) the pseudo-inverse matrix of *J* and *e* the vector representing the deviation from zero of the intracellular metabolites concentrations calculated as follows

(7) e=−R*rb'

*R* is the redundancy matrix expressed by:

(8) $$R=A_{b}-A_{n}*p_{\mathrm{inv}}\left(A_{n}\right)*A_{b}$$

And the variance-covariance matrix of the vector of residuals (*J*):

(9) $$J=R^{T}*Y_{b}*R$$

The consistency index method is based upon statistical hypothesis testing to determine whether redundancies are satisfied within the experimental error [50,51]. The first step in applying a consistency test is to determine the number of redundant equations present in the stoichiometry matrix, *A*. This is done by comparison of the number of degrees of freedom of the matrix *A* (DF=8) and the number of known measurements used in the MFA model (nine). Comparison of *h* with the χ^2^ test function determines whether the residuals of Equation 5 deviate beyond their expected distribution around 0 for a specified significance (confidence level). If a given *r* fails the consistency check (i.e., *h* > χ^2^), then there is a (confidence level)% chance that either *r* contains gross measurement errors or the assumed biochemistry is incorrect. For this, one additional redundant measurement was used as the degrees of freedom for statistical hypothesis testing. The confidence interval of the known and the unknown fluxes was analysed by calculating the estimates of the respective variance-covariance matrices (*Ψ*_*b*_ and *Ψ*_*n*_) given in Equation 10. The standard deviation vectors of the fluxes were then obtained by the squared root of the respective variance covariance matrix diagonals [52].

(10) $$\begin{array}{l} {\Psi^{\prime }}_{b}\;=1-\Psi *\mathrm{RT}{\left(R\;*\;\Psi \;*\;\mathrm{RT}\right)}^{-1}\;*\;R\;*\Psi \\ {\Psi^{\prime }}_{n}\;=\;A_{n}^{-1}\;*\;A_{b}\;*\;\Psi \;*A_{n}^{T}\;*\;{\left(A_{n}^{-1}\right)}^{T} \end{array}$$

All the computational tasks required to perform metabolic flux analysis were implemented in Matlab (version 7.12, 2011a) (MathWorks, Natick, MA).

### Experimental plan

**Figure 6:**
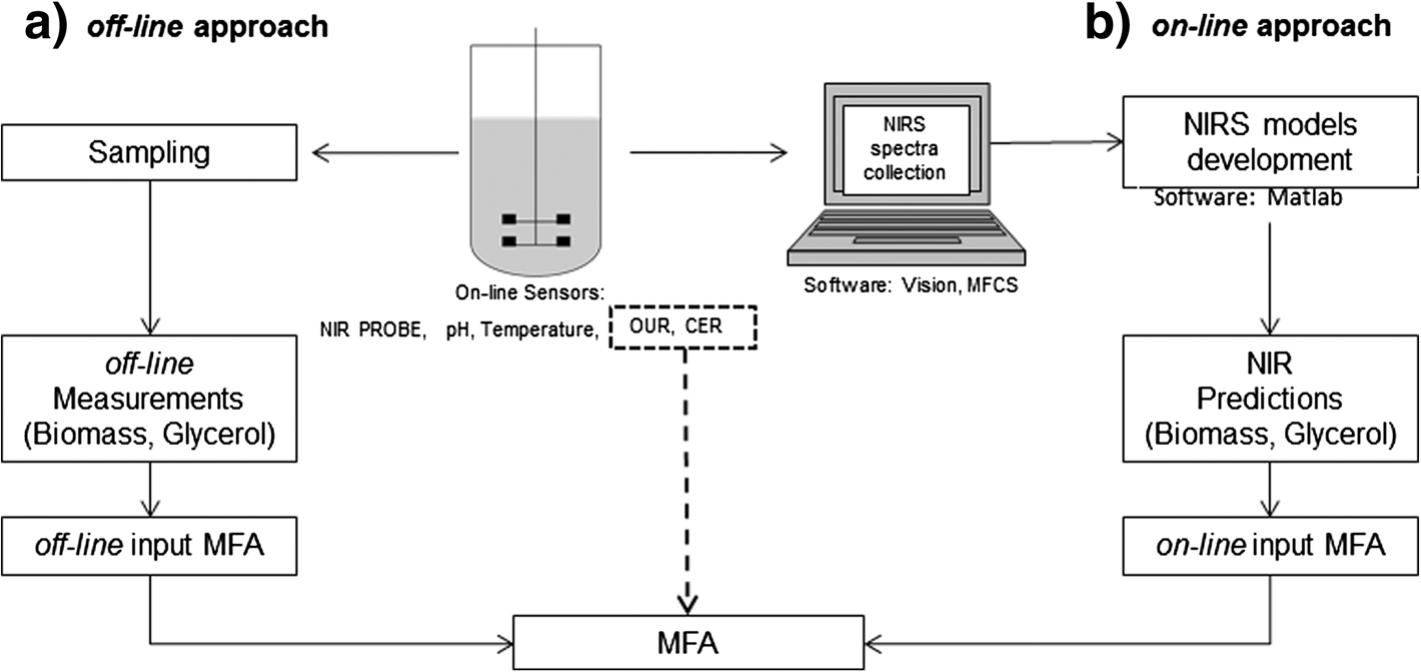
**Schematic diagram of the experimental set-up used.** Two datasets were generated **a**) off-line and **b**) on-line data and used as inputs for Metabolic Flux Analysis for the prediction of intracellular metabolites fluxes of central carbon metabolism of a chemostat culture of *Pichia pastoris*.

To obtain the estimates for the intracellular metabolic fluxes from the MFA model, extracellular rates were calculated using Equations 2 and 3 from two datasets (*off-line* and *on-line* measurements) and the results compared. A diagram of the approach used is shown in Figure 6.

## Abbreviations

AcCoA: Acetyl CoA; ACD: Acetaldehyde; ACE: Acetate; aKG: Alpha-ketoglutarate; BMGY: Buffered Glycerol-complex medium; CER: Carbon evolution rate; CO2: Carbon dioxide; Cyt: Cytosol; DCW: Dry cell weight; DHA: Dihydroxyacetone; DHAP: Dihydroxyacetone phosphate; DO: Dissolved oxygen; E4P: Erytrose-4-phosphate; ETH: Ethanol; F6P: Fructose-6-phosphate; FBP: Fructose-1,6-biphosphate; FDA: Food and Drug Administration; G6P: Glucose-6-phosphate; GAP: Glyceraldehyde-3-phosphate; GLC: Glucose; GOL: Glycerol; H: Consistency index; HCHO: Formaldehyde; ICIT: Citrate; LV: Latent variable; mABs: Monoclonal antibody; Mal: Malate; MFA: Metabolic flux analysis; Mit: Mitochondria; NIRS: Near Infrared Spectroscopy; OA: Oxaloacetate; OUR: Oxygen uptake rate; PAT: Process analytical technology; PEP: Phosphoenolpyruvate; PG3: 3-phosphoglycerate; PLS: Partial least squares; PPP: Pentose phosphate pathway; PYR: Pyruvate; R2: Correlation coefficient; R5P: Ribose-5-phosphate; RMSEC: Root-mean square error of calibration; RMSECV: Root mean square error of cross-validation; RMSEP: Root mean square error of prediction; RU5P: Ribulose-5-phosphate; S7P: Sedoheptulose-7-phosphate; SEL: Standard error of laboratory; SG: Savitzky-Golay; Suc: Succinate; TCA: tricarboxylic acid cycle; XU5P: xylulose-5-phosphate; YNB: Yeast Nitrogen Base.

## Competing interests

The authors declare that they have no competing interests.

## Authors’ contributions

MLF carried out fermentations and all the experimental procedures. MLF and AN carried out NIR modelling. JMLD and MLF carried out metabolic flux analysis. B M, LMH and DL were the supervisors and REE the co-supervisor and the coordinators of the research. All authors read and approved the final manuscript.

## Supplementary Material

Additional file 1 **Metabolic network for *P. pastoris*.** List of reactions, metabolites and stoichiometric matrix and predictions of intracellular fluxes. Click here for file
